# Comparative Genomics of *Acetobacterpasteurianus* Ab3, an Acetic Acid Producing Strain Isolated from Chinese Traditional Rice Vinegar Meiguichu

**DOI:** 10.1371/journal.pone.0162172

**Published:** 2016-09-09

**Authors:** Kai Xia, Yudong Li, Jing Sun, Xinle Liang

**Affiliations:** College of Food Science and Biotechnology, Zhejiang Gongshang University, Hangzhou, 310018, China; Fujian Agriculture and Forestry University, CHINA

## Abstract

*Acetobacter pasteurianus*, an acetic acid resistant bacterium belonging to alpha-proteobacteria, has been widely used to produce vinegar in the food industry. To understand the mechanism of its high tolerance to acetic acid and robust ability of oxidizing ethanol to acetic acid (> 12%, w/v), we described the 3.1 Mb complete genome sequence (including 0.28 M plasmid sequence) with a G+C content of 52.4% of *A. pasteurianus* Ab3, which was isolated from the traditional Chinese rice vinegar (Meiguichu) fermentation process. Automatic annotation of the complete genome revealed 2,786 protein-coding genes and 73 RNA genes. The comparative genome analysis among *A. pasteurianus* strains revealed that *A*. *pasteurianus* Ab3 possesses many unique genes potentially involved in acetic acid resistance mechanisms. In particular, two-component systems or toxin-antitoxin systems may be the signal pathway and modulatory network in *A. pasteurianus* to cope with acid stress. In addition, the large numbers of unique transport systems may also be related to its acid resistance capacity and cell fitness. Our results provide new clues to understanding the underlying mechanisms of acetic acid resistance in *Acetobacter* species and guiding industrial strain breeding for vinegar fermentation processes.

## Introduction

Vinegar is a centuries-old fermented condiment, which is produced by raw materials such as fruit, rice, grains, and cocoa. A variety of vinegars with special flavors have been populated in different regions of the world. In Italy, grape must is used as the raw material for naturally fermented Balsamic vinegar [1]. For the *kome-kurozu* vinegar manufactured in Japan, rice *koji*(moldy steamed rice grain) is used to strengthen the rice saccharification process [2]. Two types of traditional Chinese vinegar, i.e., Shanxi-aged vinegar and Zhenjiang-scented vinegar, are produced by a solid-state cultivation process using various grains as the raw material. Furthermore, Big-Qu and Small-Qu, are inoculated into the grains as starters[3]. *A*. *pasteurianus* is regarded as the dominant species in the acetic acid fermentation stage. Meiguichu vinegar, another famous type of traditional Chinese rice vinegar, is characterized by static liquid surface fermentation and complete natural microbial community without any artificial microbe inoculation in a jar (500 kiloliter volume) from spring to winter or following spring. Saccharification, alcohol fermentation and acetic acid fermentation proceed simultaneously or occur sequentially within the same jar. Currently, submerged fermentation has almost completely replaced surface fermentation technology, but the traditional high-quality vinegars such as Balsamic vinegar and Meiguichu vinegar are still produced using centuries-old empirical methods.

The major species used in vinegar production are *Acetobacter*, *Gluconobacter*, *Gluconacetobacter*, and *Komagataeibacter*[4,5]. *Komagataeibacter europaeus* (formally called *G*. *europaeus*) exhibits the highest resistance to acetic acid (10%, w/v), whereas *Gluconobacter* sp. and *A*. *pasteurianus* resist to lower acetic acid titers (~ 6%, w/v) [6]. *A*. *pasteurianus* is regarded as the dominant starter for rice vinegar fermentation in China and Japan. *A. pasteurianus* Ab3 was isolated from Meiguichu vinegar fermentation process at the Hangzhou Brewing Food Plant, China. This strain is characterized by its robust ability to oxidize ethanol to acetic acid, high acetic acid resistance, temperature adaptability, and titer yield of 12% (w/v). Previous proteome studies depicted many proteins involved in the acid resistance of *A*.*pasteurianus*[7,8]. Here, we report the high-quality complete genome sequence and annotation of strain Ab3. Among the *A*. *pasteurianus* strains, we focused especially on the genes involved in two-component systems (TCSs), toxin-antitoxin (TA) system, and membrane transporters, indicating the signaling pathway or modulatory network in response to acetic acid accumulations. The current study is a valuable supplement to the differential proteome in terms of the molecular mechanisms of acetic acid resistance in *A*. *pasteurianus*.

## Materials and Methods

### Growth conditions and genomic DNA preparation

Lyophilization was used to store *A*. *pasteurianus* Ab3 (deposited at China Center for Type Culture Collection, NO M 2013116) in the laboratory. Strain Ab3 was grown in a YPDE medium containing 10 g/L yeast extract, 5 g/ L peptone, 10 g/L D-glucose and 3% ethanol (v/v) at 30°C and 160 r/min. DNA was extracted using the TakaRaMiniBEST Universal Genomic DNA Extraction Kit (Ver.4.0, TaKaRa). The yield, purity and concentration of genomic DNA were determined using 1% agarose gel electrophoresis with λ-Hind III digest DNA Marker and PeiQing JS-6808 automatic gel imager (Bio-Rad). Approximately 80 μg of genomic DNA with a concentration of 100 ng/μL was obtained. The 16SrRNA was cloned from the isolated genomic DNA using universal bacterial primers and subsequently sequenced for phylogenetic analysis.

### Genome sequencing and assembly

The genome of *A*. *pasteurianus* Ab3 was sequenced with the next-generation sequencing platform IlluminaHiSeq 2000 (Chenglong Billion Biological Technology Co., LTD., Beijing, China). Based on the 500 bp library, the total number of reads are 2 ×11,079,017 (bp). Based on the 3 kb library database, the number of reads is 2 ×11,314,756 (bp). The genome sequence was assembled using SOAPdenovo2[9]. Subsequently, iterative optimization was performed to obtain the best K-mer using 31–85 K-mers. Both the 500 bp and 3 Kb libraries were used for building scaffolds. GapCloser was utilized to fill the holes. The number of contigs was 5. The N50 used to assemble contigs was 34.3 Kb, and the maximum length was 318 Kb. The quality of the sequencing data was estimated by calculating the quality of every read and GC content. The complete genome sequence of Ab3 was acquired via comparative analysis with other genomes of *A*. *pasteurianus*, which had been deposited and released at NCBI (National Center for Biotechnology Information, Bethesda, MD, USA) using CLCGenomicsWorkbench_6_5 software combined with PCR (polymerase chain reaction) analysis. We used Prodigal (Version 2.60) to predict genes of the genomic sequence[10]. The rRNA was identified using RNAmmer-1.2[11], while the tRNA of the genomic sequence was detected by tRNAscan-SE[12]. The predicted genes were translated and used to search the NCBI non-redundant database (NR), SwissProt[13], COG (cluster of orthologous group), GO (Gene ontology) [14], KEGG metabolic pathways[15] and InterPro[16] databases by BLASTP (Version 2.2.22). In addition, the predicted ORFs were submitted to Pfam and TMHMM (http://www.cbs.dtu.dk/services/TMHMM) for conserved domain and transmembrane domain prediction [17]. We also used PHAST [18] to predict the phage genes and the Tandem repeats finder to detect tandem repeats in the genomic sequence[19]. The insertion sequence (IS) was predicted by ISfinder (https://www-is.biotoul.fr/, *E* = 1e^-10^) [20].

### Comparative genomic analysis of *A*. *pasteurianus* Ab3

The complete acetic acid bacteria genome references used in this study were obtained from the NCBI GenBank database (S1 Table). The complete genome sequence of *A*. *pasteurianus* Ab3 was deposited at GenBank under the accession number CP012111. The comparison between the genome sequence of *A*. *pasteurianus* Ab3 and other acetic acid strains was carried out using the BRIG[21]and MAUVE programs (version 2.3.1) with default parameters[22]. CRISPR (clustered regularly interspaced short palindromic repeats) structures of complete genomes were ascertained using the CRISPRdb database[23], while the detection of CRISPRs in the draft genome sequence was completed via CRISPRfinder (http://crispr.u-psud.fr/). The putative toxin-antitoxin (TA) system, which exists in the genome sequence of Ab3, was predicted using the web-based tool TADB (http://bioinfo-mml.sjtu.edu.cn/TADB/) and RASTA-Bacteria (http://genoweb1.irisa.fr/duals/RASTA-Bacteria/). The comparative analysis of genes, including the genes involved in two-component, toxin-antitoxin, acetic acid tolerance and transport systems, was performed with Local BLAT combined with the ClustalW module of the BioEdit sequence alignment tool. The unique genes of *A*. *pasteurianus* Ab3 were either defined with a query coverage below 30% and an identity below 60%, or they did not have the most significant hits during the comparative analysis.

### Phylogenetic analysis of acetic acid bacteria using 16SrRNA and housekeeping genes

Twenty 16S rRNA sequences of acetic acid bacteria belonging to *Acetobacter*, *Gluconacetobacter*, *Gluconobacter* and *Komagataeibacter* were acquired from the NCBI database, except for *A*. *pasteurianus* Ab3. Three housekeeping genes (*dnaK*, *groeEL* and *rpoB*) were found conserved among strains of *Acetobacter*. The nucleotides sequences and amino acid sequences of these housekeeping genes were extracted from NCBI database for phylogenetic analysis. The phylogenetic tree were constructed using the MEGA 6.0 software[24]. The topological structure of the phylogenetic tree was modified using Adobe Illustrator CS5. The evolutionary history was determined using the neighbor-joining method and the Maximum Likelihood method. The phylogeny test was evaluated by bootstrap analysis (1000 replicates). All positions containing gaps and missing data were deleted.

## Results and Discussion

### General genome features of *A*. *pasteurianus* Ab3

**Fig 1:**
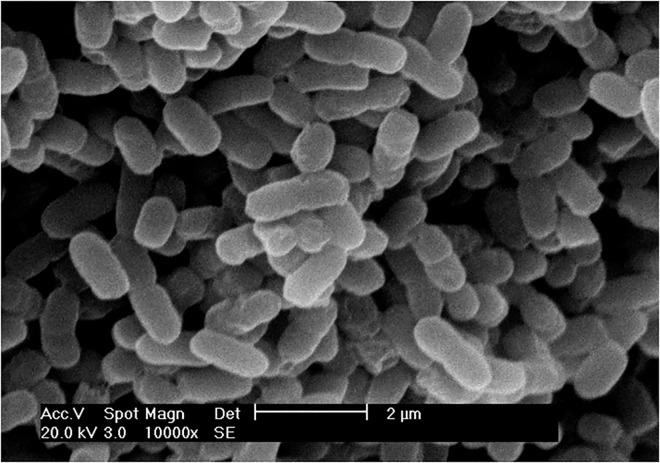
Morphology of *A*. *pasteurianus* Ab3 taken by scanning electron microscopy.

*A*. *pasteurianus* strain Ab3 is a Gram-negative, rod-shaped, acid-tolerant and acid forming strain isolated from the traditional Chinese rice vinegar (Meiguichu) fermentation process (Fig 1). The whole genome sequencing of strain Ab3 was performed with IlluminaHiSeq 2000 sequencing platforms and deposited in the GenBank database under accession number CP012111. As shown in Table 1, the size of the complete genome sequence of strain Ab3 is 3,098,045 bp, and the GC content is 52.4%. The genome sequence contains 2,981 coding sequences, 58 tRNAs and a complete 5S-23S-16S rRNA gene family. Approximately 2,786 protein-coding genes were assigned to a predicted function. The complete genome of Ab3 consists of one chromosome and six plasmids, and the size of the chromosome was smaller than that of other strains, which were mainly used in acetic acid production related to genus *Acetobacter* (Table 2). The genome sequence of strain Ab3 consists of six prophages, 23 insertion sequences, 45 tandem repeats and 157 transposases. The numerous phage genomes, insertion sequences and transposases might cause the genomic instability of Ab3, which is consistent with results published on *A*. *pasteurianus* IFO 3283–01 [25].

**Table 1:** Genome statistics of *A*. *pasteurianus* Ab3.

Attribute	Term
Genome size (bp)	3,098,045
DNA coding region (bp)	2,650,067
DNA G+C content (bp)	1,623,375
Chromosome	1
Plasmid	6
Total genes	2,981
Protein-coding genes	2,786
RNA genes	73
Pseudo genes	121
Genes with function prediction	1,583
Genes assigned to COGs	1,938
Genes with Pfam domains	2,295
Prophage	6
Insertion sequence	23
Tandem repeats sequence	45
Transposase	157
CRISPR repeats	0

**Table 2:** Comparison of sequenced genomes of *Acetobacter* strains.

Strain	a	b	c	d	e	f
Chromosome size (Mb)	2.81	2.91	2.82	3.69	3.58	2.88
GC content (%)	53.30	53	52.9	57	57.3	52.3
Protein (CDS)	2, 535	2, 644	2, 501	3, 264	3, 454	2, 408
rRNA	15	15	15	9	4	NA
tRNA	58	57	57	51	42	NA
Plasmid (Mb)	0.298	0.429	0.26	NA	NA	NA
Gene (number)	2, 688	2, 731	2, 597	3, 364	3, 528	2,414
No. of contigs	5	7	8	8	1,488	66
Status	complete	complete	complete	draft	draft	draft
Bioproject	PRJNA242487	PRJNA59279	PRJNA59279	PRJNA199175	PRJNA70715	PRJNA65823

The characters a-f represent different strains: a- *A*. *pasteurianus* Ab3; b- *A*. *pasteurianus* IFO 3283–01; c- *A*. *pasteurianus* 386B; d- *A*. *aceti* ATCC 23746; e- *A*. *acti*NBRC 14818; f- *A*. *pomorum* DM001. NA: not accessible.

**Fig 2:**
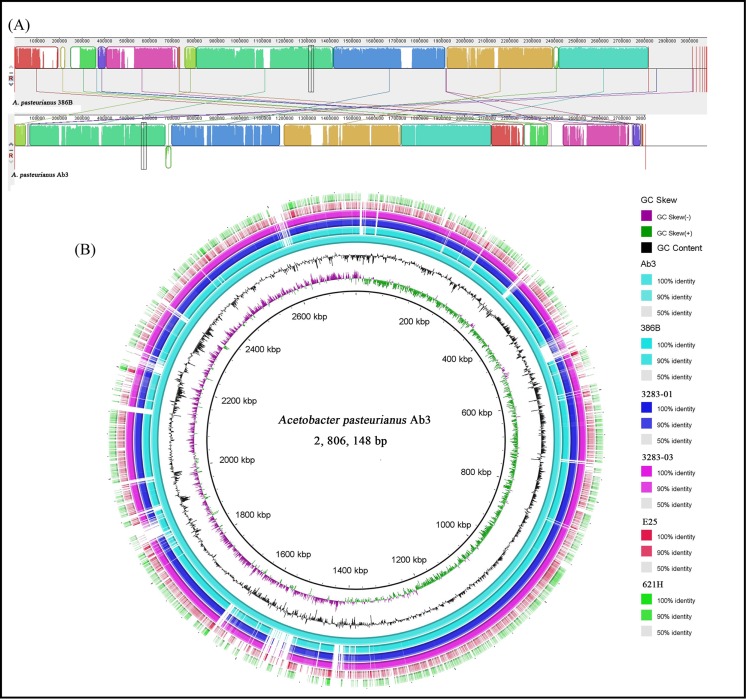
Comparative genomic analysis between *A*. *pasteurianus* Ab3 and other strains belonging to acetic acid bacteria. A: alignment of the genomes from *A*. *pasteurianus* Ab3 and 386B using MAUVE. The identically colored boxes, known as locally collinear blocks, represent homologous regions in the two sequences. The vertical lines connect the LCBs point with homologous regions between the two-genome sequences. The numbers represent the position of nucleotides. B: whole genome alignment among strain Ab3 and other strains belonging to genus *Gluconobacter* and *Komagataeibacter* using the Ab3 genome as the reference. Ab3: *A*. *pasteurianus* Ab3; 386B: *A*. *pasteurianus* 386B; 3283–01: *A*. *pasteurianus* IFO 3283–01; 3283–03: *A*. *pasteurianus* IFO 3283–03; E25: *K*. *xylinus* E25; 621H: *G*. *oxydans* 621H.

In contrast, there is less transposases and prophages in the genome than *A*. *pasteurianus*386B [26]. In strain Ab3, *dnaA* (encoding chromosomal replication initiator protein DnaA) was identified at position 1,742,348–1,743,115 bp, which differs from that of *A*. *pasteurianus* IFO-328301 (911,606–912,373 bp) and 386B (193–1,635 bp). Whole genome alignments with BRIG and MAUVE revealed the existence of numerous homologous regions, which are shared between strain Ab3 and other strains belonging to genus *Acetobacter* (Fig 2A and 2B). The CRISPR structure could not be found in the genome sequence of Ab3, which is consistent with results reported for the strain 386B genome.

### Phylogenetic and classification analysis of *A*. *pasteurianus* Ab3

**Fig 3:**
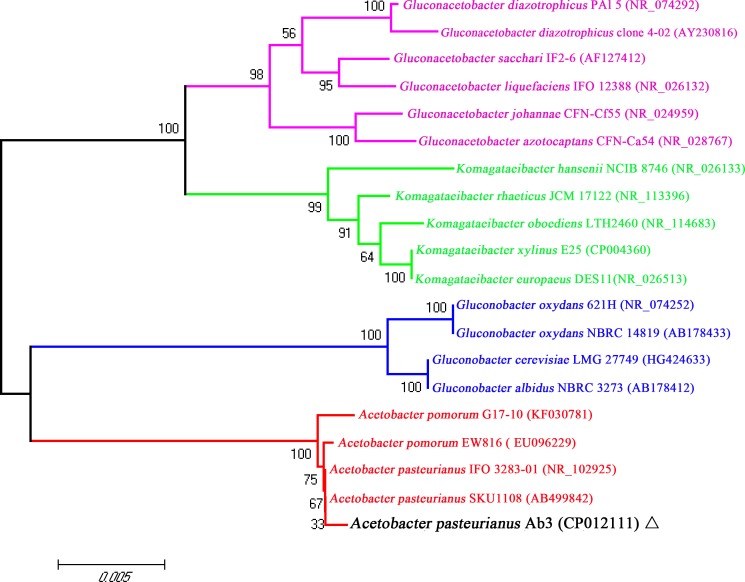
The phylogenetic tree highlights the position of *Acetobacterpasteurianus* Ab3 relative to other strains of *Acetobacter*, *Gluconacetobacter*, *Gluconobacter* and *Komagataeibacter*. The phylogenetic tree was built using 16S rRNA gene sequences aligned by CLUSTALW. Phylogenetic inferences were made using the Neighbor-joining method of the MEGA 6.0 software.

*A*. *pasteurianus* is a member of the phylum *Proteobacteria*, class *Alphaproteobacteria*, order *Rhodospirillales*, family *Acetobacteraceae*[4]. Based on 16S rRNA gene sequences from *Acetobacter*, *Gluconacetobacter*, *Komagataeibacter* and *Gluconobacter*, a phylogenetic tree was constructed with the Neighbor-Joining method. The strain Ab3 clearly clustered into the genus *Acetobacter* (Fig 3), very close to *A*. *pasteurianus* strains SKU1108 and IFO 3283-01. The sequence analysis of three housekeeping genes (*dnaK*, *groEL*, *rpoB*) additionally supports this classification, clearly indicating that strain Ab3 belongs to species *A*.*pasteurianus* (S1 and S2 Figs).

### Comparative genomics analysis and unique gene identification of *A*. *pasteurianus* Ab3

**Fig 4:**
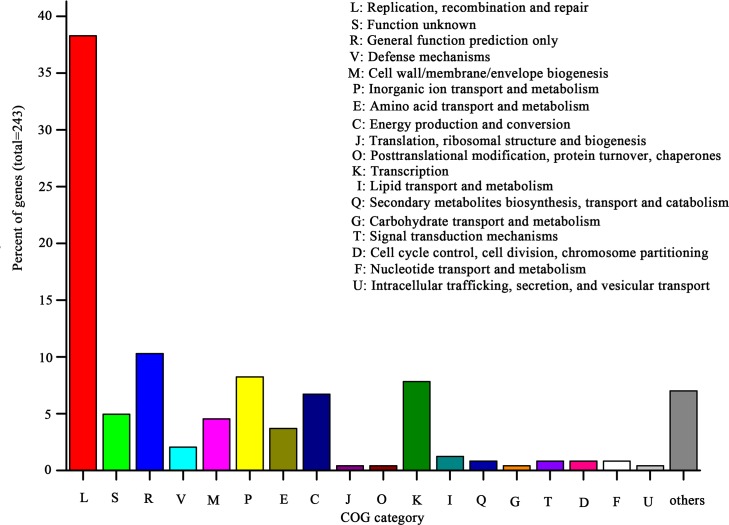
The unique genes in the genome sequence of *A*. *pasteurianus* Ab3 compared to strain 386B. The genes were categorized according to the COG annotation.

The genome of *A*.*pasteurianus* Ab3 comprises 781 unique genes including 243 genes that had been annotated according to the COG database (Fig 4). Most of the unique genes belong to categories such as replication, recombination and repair (L), inorganic ion transport and metabolism (P), transcription (K), energy production and conversion (C) and cell wall/membrane/ envelope biogenesis (M). These differences may reveal the basic functional differentiations in cell growth and metabolism.

Bacteria can adopt different signaling pathways to regulate gene expression or communicate with each other in response to biotope factors such as quorum sensing (QS). N-acylhomoserine lactones (AHL) are universally used as the signal molecule involved in the signaling system, e.g., GinI/GinRin *G*. *intermedius* (now *Komagataeibacter*), LasI/LasR in *Pseudomonas aeruginosa*, and LuxS/Isr in *Escherichia coli*[27–29]. The GinI/GinR was the first QS system reported in *Komagataeibacter* to repress the expression of *ginA* and then control acetic acid production and stress survival. However, a AHLs-dependent QS system has not been reported in any strains of genus *Acetobacter*, implying there is other signal networks sensing and modulating environmental stresses in *A*. *pasteurianus*. Notably, both two component systems (TCSs) and toxin-antitoxin system (TA) were detected in *A*. *pasteurianus* Ab3. TCSs are another type of widespread regulatory systems that respond appropriately to various stimuli such as pH, osmolality, quorum signals and nutrient starvation [30,31]. There are numerous genes encoding TCS modules in the genome sequence of strain Ab3, including 5 complete TCSs (ChvG/ChvI, KdpD/KdpE, PhoR/PhoB, NtrB/NtrC, NtrY/NtrX) and 13 genes, some of which have not been characterized (Table 3)[28]. Most of these genes are conserved in the species level with a similarity ranging from 81% to 97% when compared to strain Ab3, but the difference among genera is significant (S2 Table). The occurrence of the complete KdpD/KdpE in the plasmid sequence of strain Ab3 is unique. KdpD/KdpE has been reported to play a crucial role in integrating K^+^ homeostasis and oxidative stress signals [30,32]. In *Agrobacterium tumefaciens*, ChvG/ChvI is regarded as a global pH sensor and regulates acid-inducible genes [33,34]. ChvG/ChvI is one of the widespread TCSs among acetic acid bacteria. The average similarity of ChvG/ChvI is 92% among species of *A*. *pasteurianus*, while other strains possess homologous segments. In addition, the comparative analysis revealed that most of the TCSs in the genome sequence of strain Ab3, which were not mapped in other strains, belong to the genera *Gluconacetobacter*, *Komagataeibacter* and *Gluconobacter*, indicating the different evolution, acetic acid resistance, and regulatory network.

**Table 3:** The genes encoding TCS in the genome sequence of *A*. *pasteurianus* Ab3.

Code	Gene (ID)	Size (bp)	Product
A	AKR48263	2244	two-component hybrid sensor histidine kinase and regulator
B	AKR48409	1803	two-component sensor histidine kinase ChvG 1803
C	AKR48410	714	Transcriptional regulatory protein ChvI
D	AKR48435	690	PhoB family transcriptional regulator
E	AKR48436	1284	two-component sensor histidine kinase PhoR
F	AKR48918	2319	two-component hybrid sensor histidine kinase and regulator
G	AKR48953	732	two-component response regulator KdpE
H	AKR49048	1836	two-component sensor histidine kinase
I	AKR49049	2001	two-component hybrid sensor histidine kinase and regulator
J	AKR49998	1779	two-component hybrid sensor histidine kinase and regulator
K	AKR49254	750	two-component response regulator
L	AKR49398	252	two-component response regulator
M	AKR49486	723	two-component response regulator
N	AKR49638	723	two-component response regulator OmpR
O	AKR49677	1392	two-component response regulator NtrX
P	AKR49678	2265	two-component sensor histidine kinase NtrY
Q	AKR49679	1443	two-component response regulator NtrC
R	AKR49680	1110	two-component sensor histidine kinase NtrB
S	AKR47652	678	two-component response regulator
T	AKR49792	882	signal transduction histidine-protein kinase/phosphatase MprB
U	ALR88244	672	two-component response regulator KdpE
V	ALR88340	2688	two-component sensor histidine kinase KdpD
W	ALR88341	684	two-component response regulator KdpE

**Fig 5:**
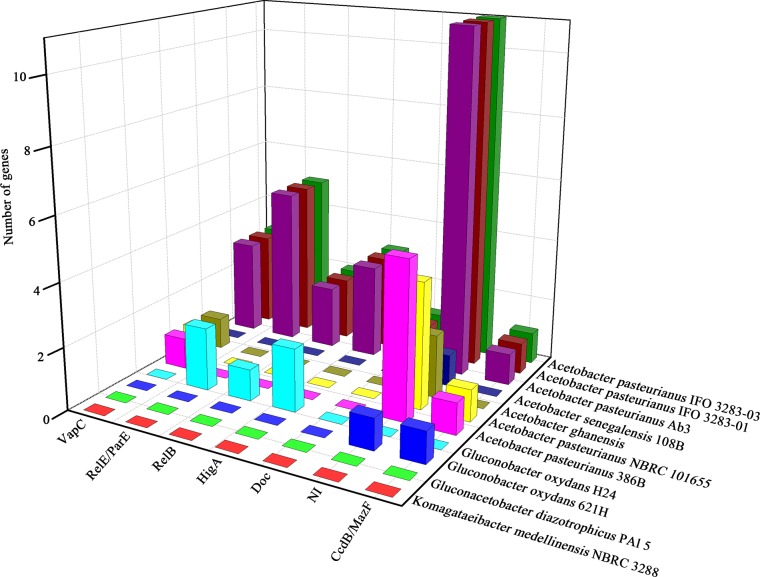
Representation of the comparative analysis of the TAs in the plasmid sequences among acetic acid bacteria using the sequence of Ab3 as a reference. The available plasmid sequences that deposited in the NCBI GenBank database involved in each strain were extracted and analyzed using Local BLAST (Blastn). Whether the TAs of Ab3 appear in other selected strains was judged according to the fact if the relative genes in other strains displayed the orthologous segments with a query coverage of 30% and identity of 60%. The distribution of the TAs between different strains were identified and illustrated in a 3D graph. X, Y and Z-axis, respectively, denote TA superfamily, organism and the number of genes.

The toxin-antitoxin system (TA) is another prevalent regulatory system that has been studied during the past decades [35,36], playing a crucial role in a wide range of important prokaryotic cellular events, including plasmid maintenance, biofilm formation, persistence, general stress response and phage defense[37,38]. However, there is few reports about TA systems in acetic acid bacteria and their functional association with acetic acid tolerance. Trcek hypothesized the possible involvement of toxin-antitoxin module, presented on plasmid of *K*. *europaeus*, in acetic acid resistance [39]. In the current study, the distribution of TA systems in acetic acid bacteria was investigated for the first time, based on genome data analysis. There are 25 putative TA systems in the genome sequence of *A*. *pasteurianus* Ab3 (including 12 pairs in chromosome and 13 pairs in plasmid sequences), which possess high reliability (score>60) determined by RASTA-Bacteria prediction (S3 and S4 Tables). Eight of the TA genes in the chromosome sequence are unique to strain Ab3 compared with other tested strains (S5 Table).The TA genes in the chromosome are relatively conserved in the species level of *A*. *pasteurianus* with a similarity ranging from 84% to 98%. In terms of CcdB/MazF, HicB, and HigA superfamily, *A*. *pasteurianus* is apparently differs from other species in the genus *Acetobacter*. Importantly, both *Gluconacetobacter* and *Gluconobacter* strains do not contain the same TA components as *Acetobacter* (Table 4). The predicted TA systems in the plasmid sequence of strain Ab3 appear more diverse in comparison with the genus *Acetobacter*, even *A*. *papsteurianus* 386B and NBRC 101655, *A*. *ghanensis*, and *A*. *senegalensis* 108B show notably different TA components (Fig 5 and S6 Table). The diversity of TA systems may indicate the different evolutionary processes or stresses, providing each strain unique genes and modulatory network. Notably, nine out of twelve toxin genes in the strain Ab3 chromosome and six out of thirteen toxin genes in its plasmid could not be identified. Hence, TA systems and especially toxin genes need more investigation.

**Table 4:** The putative TA system superfamily in the chromosome of acetic acid bacteria.

SpeciesNo^a^.	HigA^b^	CcdB/MazF^b^	HicB^b^	RelE/ParE^b^	NI^b^
1	8	2	2	1	11
2	2	2	2	0	6
3	2	2	2	0	6
4	2	2	2	0	6
5	2	2	2	0	6
6	2	2	2	0	6
7	2	2	2	0	6
8	2	2	2	0	6
9	2	2	2	0	6
10	3	2	2	0	7
11	0	0	1	0	1
12	1	1	2	0	5
13	4	2	2	0	7
14	0	0	0	0	0
15	0	0	0	0	0
16	2	2	1	0	6
17	1	0	0	0	0
18	1	0	0	0	0
19	0	0	1	0	1
20	1	0	0	0	0
21	0	0	1	0	1
22	0	0	0	0	0
23	0	0	0	0	0
24	0	0	0	0	0
25	2	2	2	0	6
26	1	0	0	0	0
27	0	0	0	0	0
28	1	0	1	0	1
29	0	0	1	0	1
30	0	0	0	0	0
31	0	0	0	0	0
32	0	0	0	0	0
33	0	0	0	0	0

^**a**^represented the same strains in S1 Table. The sequence of strain Ab3 was used as the reference.

^b^ TA amounts according to the classification by Leplae et al.[40]. The number in each column represented the existed TA modules. The detailed distributions of the TA systems in each genome (chromosome) among acetic acid bacteria were listed in S3 Table.

In addition, a variety of genes involved in transport of sulfate/sulfite, cations, nitrate/sulfate/bicarbonate, phosphate and arsenate were also compared. Accordingly, 20 out of 91 genes are unique to strain Ab3 and are involved in Fe, K^+^, Mn^2+^ and Fe^2+^ (NRAmp family) transport, silver efflux pump, Nhap/Nhad Na^+^/H^+^ and K^+^/H^+^ antiporters, nitrate transport and glutaredoxin family (Table 5). These genes may play the important roles in ion solute transport or pH homeostasis and be essential for normal cell growth under high acetic acid titer stress. Another important module is the TonB-ExbB-ExbD energy transduction system in the Ab3 genome. This system contributes the periplasmic pH homeostasis for *Helicobacter pylori* and ferric enterobactin acquisition in *Campylobacter*[41,42]. Thus, we propose that this system may act positively in internal cell pH homeostasis and acetic acid tolerance in strain Ab3.

**Table 5:** The analysis of transporters in the genome of *A*. *pasteurianus* Ab3 compared with *A*. *pasteurianus* 386B.

Category	Type	Gene amounts	Special* gene
Sulfate/Sulfite	ABC-type transport system	4	
	Related transporter	6	
Cation transport	Fe transporter	20	4
	Fe2+/Zn2+ transport system	4	
	Mg2+/Co2+ transport system	3	
	Fe3+ ABC-type transport system	4	
	Co/Zn/Cd cation transporters	2	
	K+ transport	9	5
	Mn2+ and Fe2+ (NRAmp family)	3	2
	Mn2+ and Zn2+ ABC type	2	
	Silver efflux pump	1	1
	ABC-type Metal ion transport system	4	
	Cation transport	2	
	Nhap/Nhad Na+/H+ AND K+/H+ antiporters	8	4
Nitrate/Sulfate/Bicarbonate	ABC transport system	7	
	Nitrate transporter	2	2
Phosphate	ABC type transport system	8	
Arsenate reductase and related protein	Glutaredoxin family	2	2

* representing unique gene amounts only found in the genome sequence of strain Ab3

### Genes associated with acetic acid tolerance

The most significant features of *A*. *pasteurianus* Ab3 are high acetic acid yield and resistance. Bacteria have evolved different mechanisms to cope with the deleterious effects imposed by acetic acid [43]. The mechanisms of acetic acid resistance can be roughly classified into two groups: (1) mechanisms that aim to reduce the concentration of acetic acid through catabolism and (2) mechanisms that aim to hinder the entry of acetic acid into the cell and/or suppress the deleterious effects of internal acid accumulation. S7 Table lists the genes in strain Ab3, which were previously reported to be associated with acetic acid resistance[7,8,44–47]. Strain Ab3 shares most of the transporters with other strains of *A*. *pasteurianus*. In terms of ABC transporters, strain Ab3 shows a strong similarity to *A*. *pomorum* DM001, implying different transmembrane functions from that of *A*. *pasteurianus*. Gene *glcB*, encoding malate synthase functions in acetate assimilation and energy production through glyoxylate pathway[47], is lost in strain Ab3 and helpful to the high titer accumulation (>9%, w/v) in the vinegar production.

## Conclusion

The complete genome sequence of *A*. *pasteurianus* Ab3, a strain isolated from the traditional rice vinegar (Meiguichu) fermentation process, was determined, annotated, and described in this study. Comparative genome analysis revealed that *A*. *pasteurianus* Ab3 contains several regions that are absent from *A*. *pasteurianus* 386B or IF03283-01. The occurrence of a variety of unique TCSs and TA systems in *A*. *pasteurianus* Ab3 may indicate the existence of a special signaling pathway and modulatory network coping with high acetic acid stress. The TCSs and TA systems may be a valuable alternative approach to study molecular mechanisms of acetic acid resistance in *Acetobacter*. In a nutshell, the elucidation of the complete genome sequence of strain Ab3 provides the basis for future research of both theoretic and applied vinegar fermentation.

## Supporting Information

S1 Fig Phylogenetic analysis of acetic acid bacteria belonging to genus *Acetobacter*, *Gluconacetobacter*, *Gluconobacter* and *Komagataeibacter* based on *dnak*, *groEL* and *rpoB* gene sequences. Gene sequences (complete sequences) were aligned by the CLUSTALW. Phylogenetic inferences were made using the Neighbor-joining method of the MEGA 6.0 software.
(TIF)

S2 Fig Phylogenetic analysis of acetic acid bacteria belonging to genus *Acetobacter*, *Gluconacetobacter*, *Gluconobacter* and *Komagataeibacter* based on Dnak, GroEL and RpoB protein sequences. Protein sequences (complete sequences) were aligned by the CLUSTALW. Phylogenetic inferences were made using the Neighbor-joining method of the MEGA 6.0 software.
(TIF)

S1 Table The detailed information of strains and genomes used in this study (PDF)

S2 Table The comparative analysis of two component systems among acetic acid bacteria (PDF)

S3 Table The putative toxin-antitoxin systems in the chromosome sequence of *A*. *pasteurianus* Ab3 (PDF)

S4 Table The putative toxin-antitoxin systems in plasmid sequences of *A*. *pasteurianus* Ab3 (PDF)

S5 Table The comparative analysis of toxin-antitoxin systems (in the chromosome genome sequence) among acetic acid bacteria (PDF)

S6 Table The comparative analysis of toxin-antitoxin systems (in plasmid sequences) among acetic acid bacteria (PDF)

S7 Table The comparative analysis of genes related to acetic acid tolerance between *A*. *pasteurianus* Ab3 and other acetic acid bacteria (PDF)

S8 Table The genome project information of *A*. *pasteurianus* Ab3 (PDF)
