# Supplementary figures and images for: Comparative Genomics of Acetobacterpasteurianus Ab3, an Acetic Acid Producing Strain Isolated from Chinese Traditional Rice Vinegar Meiguichu
Source: PLoS One. 2016 Sep 9;11(9):e0162172. doi: 10.1371/journal.pone.0162172 (PMC5017713; doi:10.1371/journal.pone.0162172)

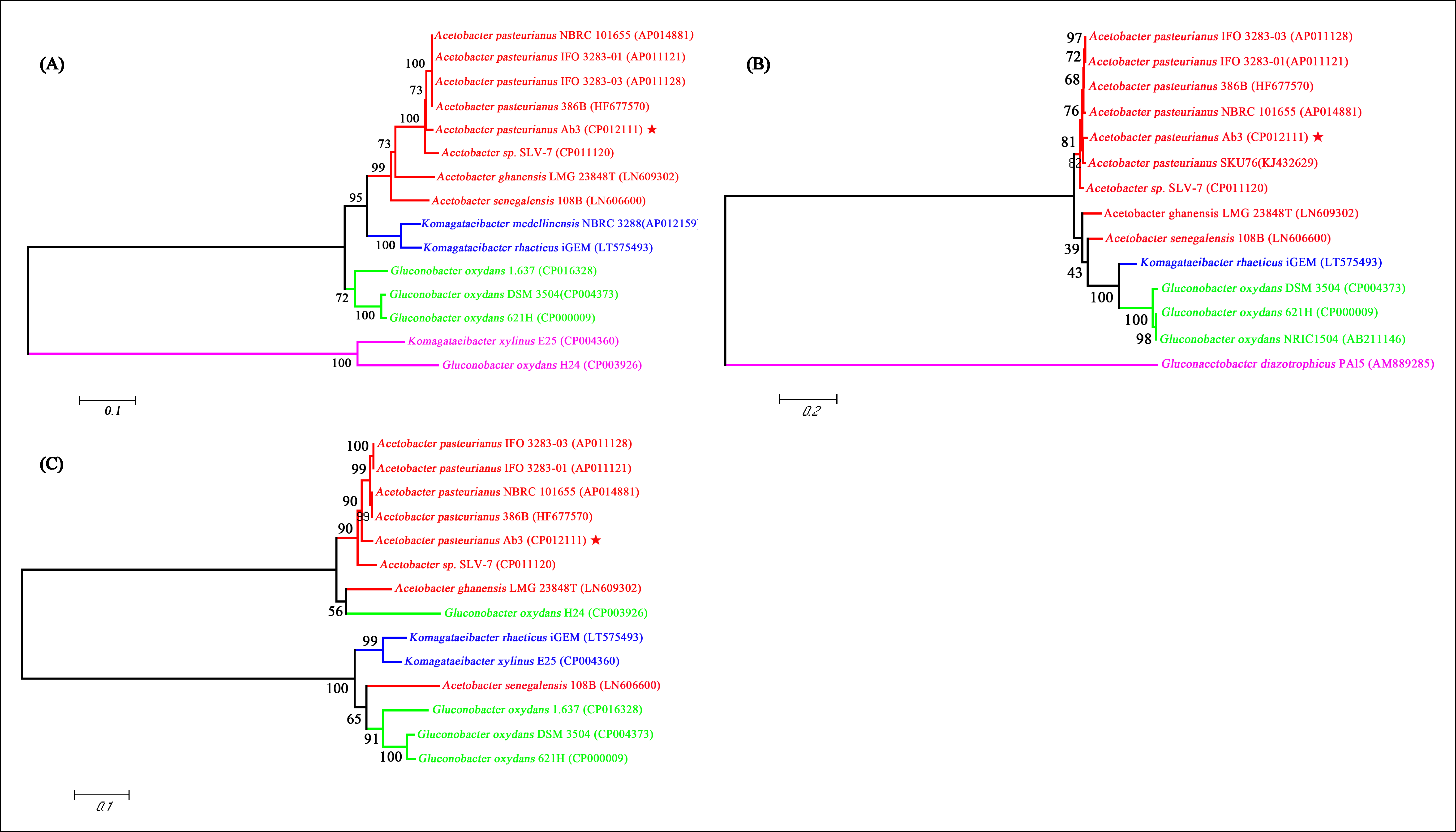

Supplement: S1 Fig — Gene sequences (complete sequences) were aligned by the CLUSTALW. Phylogenetic inferences were made using the Neighbor-joining method of the MEGA 6.0 software. (TIF) [file pone.0162172.s001.tif]

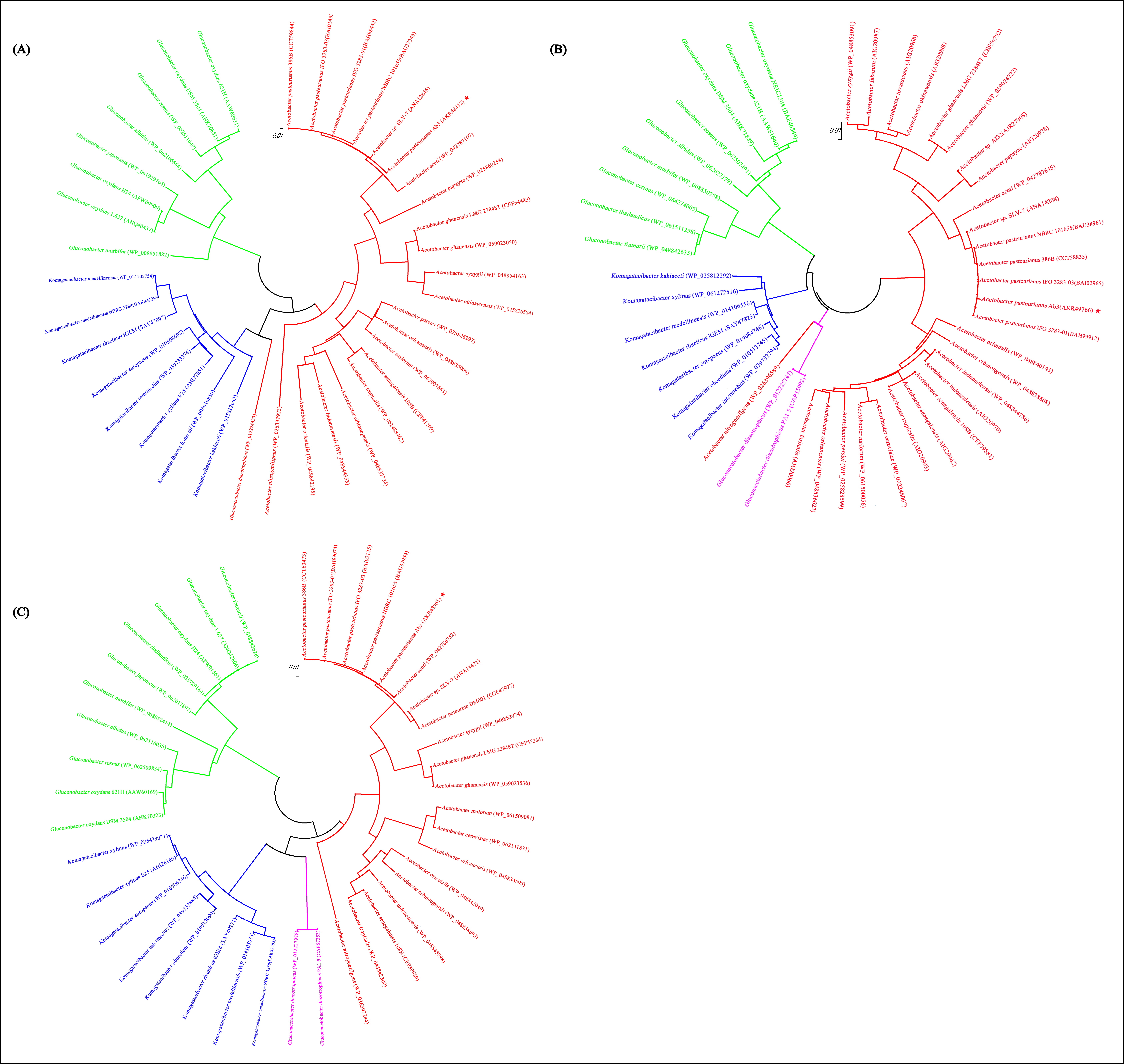

Supplement: S2 Fig — Protein sequences (complete sequences) were aligned by the CLUSTALW. Phylogenetic inferences were made using the Neighbor-joining method of the MEGA 6.0 software. (TIF) [file pone.0162172.s002.tif]
