# Supplementary material for: Comparative Genomics of Acetobacterpasteurianus Ab3, an Acetic Acid Producing Strain Isolated from Chinese Traditional Rice Vinegar Meiguichu
Source: PLoS One. 2016 Sep 9;11(9):e0162172. doi: 10.1371/journal.pone.0162172 (PMC5017713; doi:10.1371/journal.pone.0162172)
Supplement: S1 Table — (PDF) [file pone.0162172.s003.pdf]

S1 Table The detailed information of strains and genomes used in this study

Code	Organism	Accession number or project
1	*Acetobacter pasteurianus* Ab3	CP012111
2	*Acetobacter pasteurianus* IFO 3283-01	AP011121
3	*Acetobacter pasteurianus* IFO 3283-03	AP011128
4	*Acetobacter pasteurianus* IFO 3283-07	AP011135
5	*Acetobacter pasteurianus* IFO 3283-12	AP011170
6	*Acetobacter pasteurianus* IFO 3283-22	AP011142
7	*Acetobacter pasteurianus* IFO 3283-26	AP011149
8	*Acetobacter pasteurianus* IFO 3283-32	AP011156
9	*Acetobacter pasteurianus* IFO 3283-01-42C	AP011163
10	*Acetobacter pasteurianus* 386B	HF677570
11	*Acetobacter aceti* NBRC 14818	BAMU01000001
12	*Acetobacter pomorum* DM001	AEUP01000001
13	*Acetobacter pasteurianus* NBRC 101655	AP014881
14	*Acetobacter nitrogenifigens* DSM 23921	PRJNA185525
15	*Acetobacter tropicalis* NBRC 101654	PRJDA46891
16	*Acetobacter pasteurianus* IFO 3191	PRJDA65547
17	*Acetobacter papayae* JCM 25143	PRJDB553
18	*Acetobacter okinawensis* JCM 25146	PRJDB588
19	*Acetobacter persici* JCM 25330	PRJDB590
20	*Acetobacter cibinongensis* 4H-1	PRJDB503
21	*Acetobacter indonesiensis* 5H-1	PRJDB506
22	*Acetobacter orientalis* 21F-2	PRJDB507
23	*Acetobacter orleanensis* JCM 7639	PRJDB508
24	*Acetobacter syzygii* 9H-2	PRJDB509
25	*Acetobacter pasteurianus* 3P3	PRJEA61319
26	*Acetobacter ghanensis*	LN609302
27	*Acetobacter senegalensis* 108B	LN606600
28	*Acetobacter cerevisiae* LMG 1625	PRJNA288385

29	*Acetobacter malorum* LMG 1552	PRJNA288385
30	*Komagataeibacter medellinensis* NBRC 3288	NC_016027
31	*Gluconacetobacter diazotrophicus* PAl 5	NC_011365
32	*Gluconobacter oxydans* 621H	NC_006677
33	*Gluconobacter oxydans* H24	NC_019396

All the accessible plasmid sequence involved in each genome are also extracted and used in this study.
