# Supplementary material for: Comparative Genomics of Acetobacterpasteurianus Ab3, an Acetic Acid Producing Strain Isolated from Chinese Traditional Rice Vinegar Meiguichu
Source: PLoS One. 2016 Sep 9;11(9):e0162172. doi: 10.1371/journal.pone.0162172 (PMC5017713; doi:10.1371/journal.pone.0162172)
Supplement: S2 Table — (PDF) [file pone.0162172.s004.pdf]

S2 Table The comparative analysis of two component systems among acetic acid bacteria

Species No	A	B	C	D	E	F	G	H	I	J	K	L	M	N	O	P	Q	R	S	T	U	V	W
1	2244 (100%)	1803 (100%)	714 (100%)	690 (100%)	1284 (100%)	2319 (100%)	732 (100%)	1836 (100%)	2001 (100%)	1779 (100%)	750 (100%)	252 (100%)	723 (100%)	723 (100%)	1392 (100%)	2265 (100%)	1443 (100%)	1110 (100%)	678 (100%)	882 (100%)	672 (100%)	2688 (100%)	684 (100%)
2	2068 (92%)	1664 (92%)	687 (92%)	653 (94%)	1170 (91%)	2201 (94%)	683 (93%)	1735 (94%)	1864 (93%)	1655 (93%)	708 (96%)	234 (92%)	697 (96%)	676 (93%)	1318 (94%)	2176 (96%)	1342 (93%)	1026 (92%)	604 (89%)	615 (81%)	652 (97%)	2562 (95%)	628 (92%)
3	2068 (92%)	1664 (92%)	687 (92%)	653 (94%)	1170 (91%)	2201 (94%)	683 (93%)	1181 (93%)	1864 (93%)	1655 (93%)	708 (96%)	234 (92%)	697 (96%)	676 (93%)	1318 (94%)	2176 (96%)	1342 (93%)	1026 (92%)	604 (89%)	615 (81%)	652 (97%)	2562 (95%)	628 (92%)
4	2068 (92%)	1664 (92%)	687 (92%)	653 (94%)	1170 (91%)	2201 (94%)	683 (93%)	1735 (94%)	1864 (93%)	1655 (93%)	708 (96%)	234 (92%)	697 (96%)	676 (93%)	1318 (94%)	2176 (96%)	1342 (93%)	1026 (92%)	604 (89%)	615 (81%)	652 (97%)	2562 (95%)	628 (92%)
5	2068 (92%)	1664 (92%)	687 (92%)	653 (94%)	1170 (91%)	2201 (94%)	683 (93%)	1735 (94%)	1864 (93%)	1655 (93%)	708 (96%)	234 (92%)	697 (96%)	676 (93%)	1318 (94%)	2176 (96%)	1342 (93%)	1026 (92%)	604 (89%)	615 (81%)	652 (97%)	2562 (95%)	628 (92%)
6	2068 (92%)	1664 (92%)	687 (92%)	653 (94%)	1170 (91%)	2201 (94%)	683 (93%)	1181 (93%)	1864 (93%)	1655 (93%)	708 (96%)	234 (92%)	697 (96%)	676 (93%)	1318 (94%)	2176 (96%)	1342 (93%)	1026 (92%)	604 (89%)	615 (81%)	652 (97%)	2562 (95%)	628 (92%)
7	2068 (92%)	1664 (92%)	687 (92%)	653 (94%)	1170 (91%)	2201 (94%)	683 (93%)	1181 (93%)	1864 (93%)	1655 (93%)	708 (96%)	234 (92%)	697 (96%)	676 (93%)	1318 (94%)	2176 (96%)	1342 (93%)	1026 (92%)	604 (89%)	615 (81%)	652 (97%)	2562 (95%)	628 (92%)
8	2068 (92%)	1664 (92%)	687 (92%)	653 (94%)	1170 (91%)	2201 (94%)	683 (93%)	1735 (94%)	1864 (93%)	1655 (93%)	708 (96%)	234 (92%)	697 (96%)	676 (93%)	1318 (94%)	2176 (96%)	1342 (93%)	1026 (92%)	604 (89%)	615 (81%)	652 (97%)	2562 (95%)	628 (92%)
9	2067 (92%)	1664 (92%)	687 (92%)	653 (94%)	1170 (91%)	2201 (94%)	683 (93%)	1735 (94%)	1864 (93%)	1655 (93%)	708 (96%)	234 (92%)	697 (96%)	676 (93%)	1318 (94%)	2176 (96%)	1342 (93%)	1026 (92%)	604 (89%)	615 (81%)	652 (97%)	2562 (95%)	628 (92%)
10	2071 (92%)	1664 (92%)	686 (92%)	653 (94%)	1170 (91%)	2198 (94%)	685 (93%)	1734 (94%)	1864 (93%)	1655 (93%)	708 (96%)	231 (91%)	690 (96%)	677 (93%)	1319 (94%)	2181 (96%)	1344 (93%)	1034 (93%)	603 (89%)	373 (80%)	648 (96%)	-	-
11	-	-	-	-	-	-	-	-	-	-	-	-	-	-	-	-	-	-	-	-	-	-	-
12	2008 (90%)	1626(90%)	662 (92%)	628 (91%)	1138 (88%)	2091 (90%)	683 (93%)	1681(91%)	1810 (90%)	1630 (91%)	692 (92%)	232 (92%)	685 (94%)	662 (91%)	1268 (91%)	2098 (92%)	1293 (89%)	973 (88%)	598 (88%)	594 (81%)	645 (95%)	-	-
13	2071 (92%)	1664 (92%)	686 (96%)	653 (94%)	1170 (91%)	2198 (94%)	685 (93%)	1734 (94%)	1864 (93%)	1655 (93%)	708 (96%)	234 (92%)	697 (96%)	678 (93%)	1323 (95%)	2179 (96%)	1338 (92%)	1031 (92%)	601 (88%)	609 (80%)	652 (97%)	-	-
14	-	-	-	-	-	-	-	-	-	-	-	-	-	-	-	-	-	-	-	-	-	-	425 (79%)
15	-	559 (80%)	600 (80%)	-	-	-	437 (80%)	-	-	-	-	-	374 (74%)	289 (83%)	928 (79%)	871 (81%)	778 (77%)	-	312 (79%)	-	-	-	-
16	2079 (92%)	1660 (92%)	690 (76%)	651 (94%)	1179 (91%)	2199 (94%)	682 (93%)	1733 (94%)	1871 (93%)	1641 (92%)	717 (96%)	233 (92%)	705 (97%)	680 (94%)	1317 (94%)	2177 (96%)	1342 (93%)	1026 (92%)	600 (88%)	380 (82%)	626 (93%)	-	-
17	-	-	563 (80%)	-	-	-	467 (79%)	-	-	-	-	-	558 (80%)	-	1106 (80%)	803 (80%)	463 (80%)	-	-	-	-	-	-
18	-	-	584 (85%)	273 (81%)	-	-	402 (81%)	-	-	-	-	-	554 (80%)	-	1116 (81%)	817 (82%)	884 (78%)	536 (77%)	287 (78%)	-	-	1322 (82%)	532 (81%)
19	-	-	563 (83%)	-	-	-	397 (82%)	-	-	-	-	-	401 (82%)	269 (80%)	910 (79%)	741 (81%)	428 (79%)	-	293 (82%)	-	-	-	-
20	-	-	588 (82%)	-	-	-	349 (80%)	-	-	-	-	-	299 (84%)	229 (79%)	694 (78%)	861 (79%)	-	-	-	-	-	-	-
21	-	-	585 (82%)	248 (78%)	-	-	429 (79%)	-	-	-	-	-	379 (79%)	-	907 (78%)	852 (79%)	435 (78%)	-	345 (80%)	-	-	-	-
22	-	-	584 (82%)	-	-	-	368 (79%)	-	-	-	-	-	468 (81%)	-	835 (77%)	847 (77%)	-	-	-	-	-	-	-
23	-	-	589 (82%)	-	-	-	367 (82%)	-	-	-	-	-	424 (80%)	330 (78%)	1090 (78%)	837 (80%)	-	432 (79%)	339 (80%)	-	-	-	-
24	787 (80%)	642 (80%)	599 (83%)	287 (82%)	-	-	465 (81%)	-	605 (79%)	-	-	-	563 (80%)	327 (78%)	1115 (80%)	809 (83%)	911 (79%)	-	372 (81%)	-	-	-	-
25	2045 (91%)	1629 (91%)	665 (93%)	646 (93%)	1159 (90%)	2141 (92%)	695 (94%)	1693 (92%)	1834 (91%)	1632 (91%)	703 (93%)	235 (93%)	692 (95%)	674 (93%)	1289 (92%)	2134 (94%)	1333 (92%)	1029 (92%)	601 (88%)	575 (79%)	648 (96%)	-	-
26	-	764 (79%)	616 (85%)	295 (80%)	-	-	524 (79%)	-	-	-	-	-	556 (79%)	252 (80%)	1100 (79%)	1220 (81%)	901 (80%)	539 (77%)	352 (82%)	-	-	-	-
27	-	-	597 (84%)	-	-	-	443 (82%)	-	-	-	-	-	351 (77%)	286 (82%)	925 (78%)	868 (81%)	-	-	323 (79%)	-	262 (77%)	-	-
28	-	-	564 (83%)	-	-	-	368 (81%)	-	-	-	-	-	383 (80%)	268 (82%)	919 (80%)	1207 (79%)	425 (78%)	315 (81%)	329 (80%)	-	-	-	-
29	-	-	567 (83%)	-	-	-	437 (81%)	-	-	-	-	-	439 (80%)	266 (81%)	923 (80%)	864 (80%)	428 (79%)	315 (80%)	331 (80%)	-	-	-	-
30	-	-	285 (81%)	-	-	-	-	-	-	-	-	-	-	-	-	-	-	-	-	-	432 (87%)	2562 (95%)	628 (92%)
31	-	-	289 (84%)	-	-	-	-	-	-	-	-	-	-	-	-	-	-	-	-	-	-	-	-
32	-	-	-	-	-	-	-	-	-	-	-	-	-	-	-	-	-	-	-	-	-	-	-
33	-	-	-	-	-	-	-	-	-	-	-	-	-	-	-	-	-	-	-	-	-	-	-

The species number 1-33 represented the same strains used in S1 Table. The characters A-W represented the modules of two component systems existed in the genome sequence of *A. pasteurianus* Ab3. The analysis was performed using the sequence of Ab3 as the reference sequence, and the contents represented the gene size or segment (bps) that could be aligned and matched. The percent digital in the bracket represented the similarity. The mark "-" represented the absence or the relative marched sequence could not be found.
