# Supplementary material for: Comparative Genomics of Acetobacterpasteurianus Ab3, an Acetic Acid Producing Strain Isolated from Chinese Traditional Rice Vinegar Meiguichu
Source: PLoS One. 2016 Sep 9;11(9):e0162172. doi: 10.1371/journal.pone.0162172 (PMC5017713; doi:10.1371/journal.pone.0162172)
Supplement: S3 Table — (PDF) [file pone.0162172.s005.pdf]

S3 Table The putative toxin- antitoxin systems in chromosome sequence of *A. pasteurianus* Ab3

TA code	Loci (chromosome)	Hits in CDD	Type	Superfamily (name)
1-T	678865-679002	NI	Toxin	NI (HipA-1)
1-A	678583-678855	HTH_XRE [cd00093]	Antitoxin	HigA (HipB-1)
2-T	1192731-1192964	NI	Toxin	NI (HipA-2)
2-A	1192957-1193208	HTH_ XRE [pfam01381]	Antitoxin	HigA (HipB-2)
3-T	2779902-2780042	NI	Toxin	NI (HipA-3)
3-A	2779575-2779877	HTH_XRE [cd00093]	Antitoxin	HigA (HipB-3)
4-T	263195-263455	CcdB [pfam01845]	Toxin	CcdB/MazF (CcdB-1)
4-A	263464-263742	NI	Antitoxin	NI (CcdA-1)
5-T	851204-851425	NI	Toxin	NI (HipA-4)
5-A	851444-851677	HTH_XRE [smart00530]	Antitoxin	HigA (HipB-4)
6-T	875914-876255	HigB [COG3549]	Toxin	RelE/ParE (HigB-1)
6-A	876225-877346	VapI [COG3093]	Antitoxin	HigA (HigA-1)
7-T	2428931-2429071	NI	Toxin	NI (HipA-5)
7-A	2428568-2428912	HTH_XRE [cd00093]	Antitoxin	HigA (HipB-5)
8-T	2782160-2782387	NI	Toxin	NI (HipA-6)
8-A	2781807-2782187	HTH_XRE [smart00530]	Antitoxin	HigA (HipB-6)
9-T	694455-694739	NI	Toxin	NI (HicA-1)
9-A	694126-694485	HicB [COG4226]	Antitoxin	HicB (HicB-1)
10-T	960624-960830	PemK [pfam02452]	Toxin	CcdB/MazF (CcdB-2)
10-A	960457-960639	NI	Antitoxin	NI (CcdA-2)
11-T	364636-364845	NI	Toxin	NI (HicA-2)
11-A	364274-364660	HicB [COG4226]	Antitoxin	HicB (HicB-2)
12-T	1141561-1141737	NI	Toxin	NI (HipA-7)
12-A	1141774-1142268	HTH_XRE [cd00093]	Antitoxin	HigA (HipB-7)

NI: Not Identified. Hits in CDD is according to the NCBI.
