# Supplementary material for: Comparative Genomics of Acetobacterpasteurianus Ab3, an Acetic Acid Producing Strain Isolated from Chinese Traditional Rice Vinegar Meiguichu
Source: PLoS One. 2016 Sep 9;11(9):e0162172. doi: 10.1371/journal.pone.0162172 (PMC5017713; doi:10.1371/journal.pone.0162172)
Supplement: S4 Table — (PDF) [file pone.0162172.s006.pdf]

S4 Table The putative toxin- antitoxin systems in plasmid sequences of *A. pasteurianus* Ab3

TA code	Size (aa)	Hits in CDD	Type	Superfamily (name)
1-T	85	VagC [COG4456]	Toxin	NI (Vagc-1)
1-A	136	VapC [COG1487]	Antitoxin	VapC (VapC-1)
2-T	84	VagC [COG4456]	Toxin	NI (VagC-2)
2-A	136	VapC [COG1487]	Antitoxin	VapC (VapC-2)
3-T	97	RelE [COG2026]	Toxin	RelE/ParE (RelE-1)
3-A	86	RelB [pfam04221]	Antitoxin	RelB (RelB-1)
4-T	78	VagC [COG4456]	Toxin	NI (VagC-3)
4-A	137	VapC [COG1487]	Antitoxin	VapC (VapC-3)
5-T	100	RelE [COG2026]	Toxin	RelE/ParE (RelE-2)
5-A	112	RelB [pfam04221]	Antitoxin	RelB (RelB-2)
6-A	112	RelE [COG2026]	Toxin	RelE/ParE (HigB-1)
6-A	99	HTH_XRE [cd00093]	Antitoxin	HigA (HigA-1)
7-T	59	NI	Toxin	NI (HigB-2)
7-A	98	HTH_XRE [cd00093]	Antitoxin	HigA (HigA-2)
8-T	151	NI	Toxin	RelE/ParE (HigB-3)
8-A	98	HTH_XRE [cd00093]	Antitoxin	HigA (HigA-3)
9-T	165	NI	Toxin	NI (AbrA-1)
9-A	136	AbrB [COG2002]	Antitoxin	NI (AbrB-1)
10-T	56	NI	Toxin	NI (HTI-1)
10-A	146	HTH_4 [pfam01402]	Antitoxin	NI (HTH-1)
11-T	64	Doc [COG3654]	Toxin	Doc (Doc-1)
11-A	37	NI	Antitoxin	NI (DoA-1)
12-T	173	MazF [COG2337]	Toxin	CcdB/MazF (MazF-1)
12-A	134	NI	Antitoxin	NI (MazE-1)
13-T	57	RelE [COG2026]	Toxin	RelE/ParE (RelE-3)
13-A	52	NI	Antitoxin	NI (RelB-3)

NI: Not Identified. Hits in CDD is according to the NCBI.
