# Supplementary material for: Comparative Genomics of Acetobacterpasteurianus Ab3, an Acetic Acid Producing Strain Isolated from Chinese Traditional Rice Vinegar Meiguichu
Source: PLoS One. 2016 Sep 9;11(9):e0162172. doi: 10.1371/journal.pone.0162172 (PMC5017713; doi:10.1371/journal.pone.0162172)
Supplement: S5 Table — (PDF) [file pone.0162172.s007.pdf]

S5 Table The comparative analysis of toxin-antitoxin systems (in chromosome genome sequence) among acetic acid bacteria

Species No*	1-T[a]	1-A[b]	2-T[a]	2-A[b]	3-T[a]	3-A[b]	4-T[a]	4-A[b]	5-T[a]	5-A[b]	6-T[a]	6-A[b]	7-T[a]	7-A[b]	8-T[a]	8-A[b]	9-T[a]	9-A[b]	10-T[a]	10-A[b]	11-T[a]	11-A[b]	12-T[a]	12-A[b]
1	138(100%)	273(100%)	234(100%)	252(100%)	141(100%)	303(100%)	261(100%)	279(100%)	222(100%)	234(100%)	342(100%)	1112(100%)	141(100%)	345(100%)	228(100%)	381(100%)	285(100%)	360(100%)	207(100%)	183(100%)	210(100%)	387(100%)	177(1000%)	495(100%)
2	-	-	-	-	-	-	138(84%)	237(87%)	88(98%)	-	-	-	-	-	-	322(84%)	271(95%)	356(98%)	198(95%)	171(93%)	196(95%)	312(90%)	152(87%)	423(87%)
3	-	-	-	-	-	-	138(84%)	237(87%)	88(98%)	-	-	-	-	-	-	322(84%)	271(95%)	356(98%)	198(95%)	171(93%)	196(95%)	312(90%)	152(87%)	423(87%)
4	-	-	-	-	-	-	138(84%)	237(87%)	88(98%)	-	-	-	-	-	-	322(84%)	271(95%)	356(98%)	198(95%)	171(93%)	196(95%)	312(90%)	152(87%)	423(87%)
5	-	-	-	-	-	-	138(84%)	237(87%)	88(98%)	-	-	-	-	-	-	322(84%)	271(95%)	356(98%)	198(95%)	171(93%)	196(95%)	312(90%)	152(87%)	423(87%)
6	-	-	-	-	-	-	138(84%)	237(87%)	88(98%)	-	-	-	-	-	-	322(84%)	271(95%)	356(98%)	198(95%)	171(93%)	196(95%)	312(90%)	152(87%)	423(87%)
7	-	-	-	-	-	-	138(84%)	237(87%)	88(98%)	-	-	-	-	-	-	322(84%)	271(95%)	356(98%)	198(95%)	171(93%)	196(95%)	312(90%)	152(87%)	423(87%)
8	-	-	-	-	-	-	138(84%)	237(87%)	88(98%)	-	-	-	-	-	-	322(84%)	271(95%)	356(98%)	198(95%)	171(93%)	196(95%)	312(90%)	152(87%)	423(87%)
9	-	-	-	-	-	-	138(84%)	237(87%)	88(98%)	-	-	-	-	-	-	322(84%)	271(95%)	356(98%)	198(95%)	171(93%)	196(95%)	312(90%)	152(87%)	423(87%))
10	-	-	-	-	112(96%)	278(91%)	137(83%)	237(87%)	87(97%)	-	-	-	-	-	222(97%)	338(90%)	-	348(96%)	198(95%)	171(93%)	198(95%)	314(90%)	152(87%)	407(88%)
11	-	-	-	-	-	-	-	-	-	-	-	-	-	-	-	-	205(80%)	173(82%)	-	-	-	-	-	-
12	-	-	-	-	-	-	-	-	107 (96%)	-	-	-	-	-	-	-	272(95%)	336(93%)	185(89%)	156(86%)	167(92%)	307(89%)	124(83%)	424(87%)
13	-	256(98%)	-	-	68(98%)	278(91%)	138(84%)	237(87%)	113(94%)	-	-	-	-	-	-	311(84%)	273(95%)	343(96%)	198(95%)	171(93%)	196(95%)	312(90%)	152(87%)	424(87%)
14	-	-	-	-	-	-	-	-	-	-	-	-	-	-	-	-	-	-	-	-	-	-	-	-
15	-	-	-	-	-	-	-	-	-	-	-	-	-	-	-	-	-	-	-	-	-	-	-	-
16	-	-	-	-	105 (92%)	-	138(84%)	233(86%))	-	-	-	-	-	-	226(99%)	360(94%)	-	-	198(95%)	171(93%)	195(94%)	289(92%)	154(89%)	427(88%)
17	-	-	-	-	-	-	-	-	-	-	-	-	-	-	-	-	-	-	-	-	-	-	-	124(83%)
18	-	-	-	-	-	-	-	-	-	-	-	-	-	-	-	-	-	-	-	-	-	-	-	152(79%)
19	-	-	-	-	-	-	-	-	-	-	-	-	-	-	-	-	216(84%)-	302(85%)	-	-	-	-	-	-
20	-	-	-	-	-	-	-	-	-	-	-	-	-	-	-	-	-	-	-	-	-	-	-	114 (83%)
21	-	-	-	-	-	-	-	-	-	-	-	-	-	-	-	-	236(83%)	300(84%)	-	-	-	-	-	-
22	-	-	-	-	-	-	-	-	-	-	-	-	-	-	-	-	-	-	-	-	-	-	-	-
23	-	-	-	-	-	-	-	-	-	-	-	-	-	-	-	-	-	-	-	-	-	-	-	-
24	-	-	-	-	-	-	-	-	-	-	-	-	-	-	-	-	-	-	-	-	-	-	-	-
25	-	-	-	-	-	-	151(84%)	241(88%)	88(98%)	-	-	-	-	-	-	306(81%)	283(99%)	346(97%)	184(89%)	160(87%)	193(93%)	347(89%)	150(85%)	423(87%)
26	-	-	-	-	-	-	-	-	-	-	-	-	-	-	-	-	-	-	-	-	-	-	-	155(79%)
27	-	-	-	-	-	-	-	-	-	-	-	-	-	-	-	-	-	-	-	-	-	-	-	-
28	-	-	-	-	-	-	-	-	-	-	-	-	-	-	-	-	215(84%)	300(84%)	-	-	-	-	-	121(82%)
29	-	-	-	-	-	-	-	-	-	-	-	-	-	-	-	-	241(84%)	302(85%)	-	-	-	-	-	-
30	-	-	-	-	-	-	-	-	-	-	-	-	-	-	-	-	-	-	-	-	-	-	-	-
31	-	-	-	-	-	-	-	-	-	-	-	-	-	-	-	-	-	-	-	-	-	-	-	-
32	-	-	-	-	-	-	-	-	-	-	-	-	-	-	-	-	-	-	-	-	-	-	-	-
33	-	-	-	-	-	-	-	-	-	-	-	-	-	-	-	-	-	-	-	-	-	-	-	-

* represented the same strains in S1 Table. [a] and [b] represented toxin and antitoxin gene size (bp) and similarity (%) respectively, using the modules of toxin-antitoxin systems in the genome of *A. pasteurianus* Ab3 as the reference sequence. The mark "-" represents absence or the relative marched sequence in other genome sequence could not be found.
