# Supplementary material for: Comparative Genomics of Acetobacterpasteurianus Ab3, an Acetic Acid Producing Strain Isolated from Chinese Traditional Rice Vinegar Meiguichu
Source: PLoS One. 2016 Sep 9;11(9):e0162172. doi: 10.1371/journal.pone.0162172 (PMC5017713; doi:10.1371/journal.pone.0162172)
Supplement: S6 Table — (PDF) [file pone.0162172.s008.pdf]

S6 Table The comparative analysis of toxin-antitoxin systems (in plasmid sequences) among acetic acid bacteria

TA code	1*	2*	3*	10*	13*	26*	27*	30*	31*	32*	33*
1-T[a]	258(100%)	229(88%)	229(88%)	227(88%)	227(88%)	-	-	-	-	-	-
1-A[b]	411(100%)	246(85%)	246(85%)	352(87%)	352(87%)	156(77%)	-	-	-	-	-
2-T[a]	255(100%)	255(100%)	255(100%)	-	-	-	-	-	-	-	-
2-A[b]	411(100%)	411(100%)	411(100%)	-	-	-	-	-	-	-	-
3-T[a]	294(100%)	294(100%)	294(100%)	-	-	-	-	-	-	-	264(89%)
3-A[b]	261(100%)	261(100%)	261(100%)	-	-	-	-	-	-	-	236(90%)
4-T[a]	237(100%)	237(100%)	237(100%)	-	-	-	-	-	-	-	-
4-A[b]	414(100%)	335(99%)	335(99%)	-	-	-	-	-	-	-	-
5-T[a]	303(100%)	303(100%)	303(100%)	-	-	-	-	-	-	-	-
5-A[b]	339(100%)	330(97%)	330(97%)	-	-	-	-	-	-	-	-
6-A[b]	300(100%)	298(99%)	298(99%)	-	-	-	-	-	-	-	-
6-T[a]	339(100%)	213(98%)	213(98%)	-	-	-	-	-	-	-	-
7-A[b]	297(100%)	297(100%)	297(100%)	-	-	-	-	-	-	-	244(82%)
7-T[a]	180(100%)	173(96%)	173(96%)	-	-	79(97%)	-	-	-	-	-
8-A[b]	297(100%)	297(100%)	297(100%)	-	-	-	-	-	-	-	244(82%)
8-T[a]	456(100%)	456(100%)	456(100%)	-	-	-	-	-	-	-	293(85%)
9-A[b]	411(100%)	411(100%)	411(100%)	392(97%)	392(97%)	-	-	-	-	-	-
9-T[a]	498(100%)	490(98%)	490(98%)	475(95%)	478(95%)	-	-	-	-	-	-
10-A[b]	441(100%)	438(99%)	438(99%)	-	-	-	-	-	-	-	-
10-T[a]	171(100%)	170(99%)	170(99%)	87(87%)	87(87%)	-	-	-	-	103(82%)	-
11-T[a]	195(100%)	192(98%)	192(98%)	-	-	-	-	-	-	-	-
11-A[b]	114(100%)	114(100%)	114(100%)	-	-	-	-	-	-	-	-
12-T[a]	522(100%)	519(99%)	519(99%)	273(88%)	273(88%)	-	-	-	-	344(80%)	-
12-A[b]	405(100%)	300(99%)	300(99%)	136(95%)	-	138(97%)	127 (90%)	-	-	-	-

13-T[a]	174(100%)	171(98%)	171(98%)	-	-	-	-	-	-	-	-
13-A[b]	159(100%)	156(98%)	156(98%)	-	-	-	-	-	-	-	-

[*]represented the same strains in S1 Table. [a] and [b] represented toxin and antitoxin gene size (bp) and similarity (%) respectively, using the modules of toxin-antitoxin systems in the plasmids of *A. pasteurianus* Ab3 as the reference sequence. The mark "-" represents absence or the relative marched sequence in other genome sequence could not be found.
