# Supplementary material for: Comparative Genomics of Acetobacterpasteurianus Ab3, an Acetic Acid Producing Strain Isolated from Chinese Traditional Rice Vinegar Meiguichu
Source: PLoS One. 2016 Sep 9;11(9):e0162172. doi: 10.1371/journal.pone.0162172 (PMC5017713; doi:10.1371/journal.pone.0162172)
Supplement: S7 Table — (PDF) [file pone.0162172.s009.pdf]

S7 Table The comparative analysis of genes related to acetic acid tolerance of between *A. pasteurianus* Ab3 and other acetic acid bacteria.

gene	product	organism	size(bp)	identity
aarA	citrate synthase	*A.pasteurianus* 386B	1311	97%
		A.pasteurianus Ab3	1307	
aarC	Succinyl-COA:acetate COA transferase	*A.pasteurianus* 386B	1518	96%
		A.pasteurianus Ab3	1518	
grpE	GrpE protein	*A.pasteurianus* 3283	597	96%
		A.pasteurianus Ab3	597	
dnaK	Chaperone protein DnaK	*A.pasteurianus*	1905	97%
		A.pasteurianus Ab3	1905	
dnaJ	Chaperone protein DnaJ	*A.pasteurianus*	1143	96%
		A.pasteurianus Ab3	1143	
groS	10 kDa chaperonin	*A.pasteurianus* IFO 3283	372	97%
		A.pasteurianus Ab3	370	
groL	60 kDa chaperonin	*A.pasteurianus* IFO 3283	1641	97%
		A.pasteurianus Ab3	1641	
trx	ubiquinone oxidase	*Streptomyces venezuelae*	1743	NO
		A.pasteurianus Ab3		
uvrB	UvrABC system protein B	*A.pasteurianus* 386B	2247	93%
		A.pasteurianus Ab3	2241	
uvrC	UvrABC system protein C	*A.pasteurianus* 386B	1914	93%
		A.pasteurianus Ab3	1904	
clpB	Endopeptidase Clp ATP-binding chain B	*A.pasteurianus* 386B	2616	96%
		A.pasteurianus Ab3	2616	
aceA	Isocitrate lyase	*Acetobacter aceti* 1023	348	95%
		A.pasteurianus Ab3	2X348	
glcB	Malate synthase A	*Acetobacter aurantius*	1614	NO
		A.pasteurianus Ab3		
cydD	ABC transporter ATP-binding protein cydD	*A.pasteurianus* IFO 3283	1689	86%
		A.pasteurianus Ab3	1688	
APA386B_1P127	ABC transporter ATP-binding protein	*A.pasteurianus* 386B	1734	NO
		A.pasteurianus Ab3		
cydC	ABC transporter, CydDC cysteine exporter	*A.pasteurianus* 386B	1692	88%
		A.pasteurianus Ab3	1686	
APA03_13300	ABC transporter ATP-binding protein	*A.pasteurianus* IFO 3283	1761	NO
		A.pasteurianus Ab3		
APA386B_1700	Putative ABC transporter ATP-binding protein	*A.pasteurianus* 386B	1680	95%
		A.pasteurianus Ab3	1673	
yddA	Putative ABC transporter ATP-binding protein	*A.pomorum* DM001	1734	NO
		A.pasteurianus Ab3		

taub	Putative ABC transporter ATP-binding protein	*A.pomorum* DM001<br>*A.pasteurianus* Ab3	1356<br>1356	89%
yhiH	Putative ABC transporter ATP-binding protein	*A.pomorum* DM001<br>*A.pasteurianus* Ab3	2736<br>2728	90%
yjjK	Putative ABC transporter ATP-binding protein	*A.pomorum* DM001<br>*A.pasteurianus* Ab3	1680<br>1680	93%
msbA	Putative ABC transporter ATP-binding protein	*A.pomorum* DM001<br>*A.pasteurianus* Ab3	1851<br>1851	90%
proV	Putative ABC transporter ATP-binding protein	*A.pomorum* DM001<br>*A.pasteurianus* Ab3	819<br>819	90%
YheS	Putative ABC transporter ATP-binding protein	*A.pomorum* DM001<br>*A.pasteurianus* Ab3	1623<br>1623	87%
yhbg	Putative ABC transporter ATP-binding protein	*A.pomorum* DM001<br>*A.pasteurianus* Ab3	828<br>824	90%
cysA	Putative ABC transporter ATP-binding protein	*A.pomorum* DM001<br>*A.pasteurianus* Ab3	792<br>791	89%
ssuC	Putative ABC transporter ATP-binding protein	*A.pomorum* DM001<br>*A.pasteurianus* Ab3	783<br>783	92%
tauc	Putative ABC transporter permease protein	*A.pomorum* DM001<br>*A.pasteurianus* Ab3	768<br>719	87%
yvrB	Putative ABC transporter permease protein	*A.pomorum* DM001<br>*A.pasteurianus* Ab3	1014<br>1010	89%
fecD	Putative ABC transporter permease protein	*A.pomorum* DM001<br>*A.pasteurianus* Ab3	1119<br>1119	87%
yrbE	Putative ABC transporter permease protein	*A.pomorum* DM001<br>*A.pasteurianus* Ab3	1158<br>1158	88%
AmDm 5_2882	Putative ABC transporter membrane protein	*Acetobacter malorum*<br>*A.pasteurianus* Ab3	717	NO
aatA	ABC transporter	*Acetobacter aceti*<br>*A.pasteurianus* Ab3	1776<br>1786	86%
AmDm 5_2815	Putative ABC transporter permease	*Acetobacter malorum*<br>*A.pasteurianus* Ab3	2472	NO

The identity represents similarity between each gene that compared. NO: NO means that the gene listed was not found in the genome sequence of Ab3.
