# Supplementary material for: Comparative Genomics of Acetobacterpasteurianus Ab3, an Acetic Acid Producing Strain Isolated from Chinese Traditional Rice Vinegar Meiguichu
Source: PLoS One. 2016 Sep 9;11(9):e0162172. doi: 10.1371/journal.pone.0162172 (PMC5017713; doi:10.1371/journal.pone.0162172)
Supplement: S8 Table — (PDF) [file pone.0162172.s010.pdf]

S8 Table The genome project information of *A. pasteurianus* Ab3

Attribute	Term
Finishing quality	High-quality draft
Libraries used	One pair-end 500 bp library and one pair-end 3 Kb library
Sequencing platforms	Illumina HiSeq 2000
Fold coverage	500 x (based on 500 bp and 3 Kb library)
Assemblers	SOAPdenovo
Gene calling method	Prodigal
Genbank ID	CP012111
Genbank Date of Release	July 23, 2015
GOLD ID	Gi0072517
BIOPROJECT	PRJNA242487
Project relevance	Quorum sensing & signaling pathway & food industry
Source Material Identifier	CCTCC NO: M 2013116
